# A Novel Use of Liposomal Bupivacaine in Erector Spinae Plane Block for Pediatric Congenital Cardiac Surgery

**DOI:** 10.1155/2021/5521136

**Published:** 2021-03-12

**Authors:** Stylianos Voulgarelis, Gregory M. Halenda, Justinn M. Tanem

**Affiliations:** ^1^Department of Anesthesiology, Division of Pediatric Anesthesiology, Children's of Wisconsin, Medical College of Wisconsin, Milwaukee, WI, USA; ^2^Department of Pediatrics, Division of Pediatric Critical Care, Children's of Wisconsin, Medical College of Wisconsin, Milwaukee, WI, USA

## Abstract

We describe the use of liposomal bupivacaine (Exparel) in erector spinae plane blocks for two patients undergoing pediatric cardiac surgery with cardiopulmonary bypass and one undergoing division of the compressive vascular ring. The perioperative course of all patients was remarkable for low pain and sedation scores, especially after chest tube removal. Erector spinae plane blocks are an expanding pain-control technique in both adult and pediatric cardiac surgery for postoperative analgesia. Liposomal bupivacaine offers prolonged analgesia and may be an attractive option for this indication.

## 1. Introduction

Erector spinae plane block (ESPB) is an emerging technique for pain management in a variety of pediatric surgeries [1]. The ESPB is a particularly interesting pain technique as it is technically easy to learn and perform, is less invasive than a paravertebral or epidural block, and may provide effective analgesia for a variety of thoracic and abdominal procedures. The ESPB has been studied in both adult [2, 3] and pediatric [4–7] cardiac surgery and generally found to be safe, effective, and expedient. The use of liposomal bupivacaine in an ESPB may significantly prolong the effect of the pain control as compared to plain bupivacaine. We present reports of three pediatric cardiac surgical patients who received ESPB using liposomal bupivacaine. In all cases, blocks were well tolerated with no signs of toxicity or adverse effects. None of the patients required additional opioids after chest tube removal. The patients' parents provided written consent for publication.

## 2. Cases Presentation

### 2.1. Case 1

A 10-month-old 7.9 kg female presented for bidirectional Glenn palliation surgery subsequent to a complex cardiac history including heterotaxy, hypoplastic left ventricle, severely unbalanced atrioventricular (AV) canal, right-dominant double-outlet right ventricle, and moderate-to-severe pulmonary stenosis. After induction of anesthesia, bilateral thoracic level 7 (T7) ESPB were performed using an admixture of 2 ml of 1.33% liposomal bupivacaine (about 3.4 mg/kg) and 6 ml of 0.25% plain bupivacaine, split evenly for each side. The Glenn procedure and PA band placement were performed on cardiopulmonary bypass at 32°C. The patient remained intubated through postoperative day (POD) zero for concerns of bleeding at the Glenn anastomosis. Overnight POD#0, the patient was sedated with dexmedetomidine infusion at 0.6 mcg/kg/hr and received four doses of intravenous morphine at 0.1 mg/kg (Tables 1 and 2). She was extubated on POD#1 in the morning and received 3 additional doses of morphine at 0.1 mg/kg through POD#1. On POD#2, the chest tubes were removed and no additional opioids, neither intravenous (IV) nor oral, were administered through discharge. She remained playful through hospitalization with no concerns of discomfort, and parents had no concerns regarding pain control.

### 2.2. Case 2

A 7-year-old 27.3 kg boy with past medical history significant for autism, horseshoe kidney, and right ventricle to pulmonary artery (RV-PA) conduit dysfunction presented for conduit replacement under cardiopulmonary bypass. A complex cardiac past medical history included neonatal critical aortic stenosis status after neonatal balloon valvotomy of the aortic valve and further repair by Ross–Konno aortic homograft with RV-PA conduit. After induction of anesthesia, bilateral bilevel (T5 and T8) ESPBs were performed using an admixture of total 8 ml liposomal bupivacaine (about 3.9 mg/kg) and 12 ml of 0.375% bupivacaine. In this patient, bilevel (T5 and T8) blocks were chosen to ensure lower thoracic dermatomal coverage all the way to the insertion site of the chest tubes and the mediastinal drain. Local anesthetic was divided evenly across four injection sites. The RV-PA conduit change was performed on cardiopulmonary bypass at 34°C. Surgery was uneventful, and the patient was extubated in the operating room and transferred to the ICU on minimal vasoactive support (milrinone infusion 0.5 mcg/kg/min, epinephrine infusion 0.01 mcg/kg/min). On POD#0, he was sedated with dexmedetomidine infusion at 0.6 mcg/kg/hr. Through POD#0, he received several doses of IV morphine totaling 0.46 mg/kg, and on POD#1, he received 0.28 mg/kg of morphine total (Tables 1 and 2). Chest tubes were removed the morning of POD#2, and no additional opioids, neither IV nor PO, were required through hospitalization. Parents had no concerns regarding pain control.

### 2.3. Case 3

An 8-year-old 19.7 kg boy presented for thoracotomy for division of the compressive vascular ring. Past medical history was complex including Ebstein anomaly, chromosome 13 deletion, CHARGE syndrome, bilateral choanal atresia, systemic hypertension, and Dandy–Walker malformation. After induction of anesthesia, the patient underwent a unilateral, bilevel (T5 and T7) ESPB. Drug admixture included 6 ml of 1.33% liposomal bupivacaine (about 4 mg/kg) with 4 ml of 0.5% bupivacaine. The patient underwent an uneventful procedure and was extubated at the conclusion of surgery. He was maintained on dexmedetomidine infusion at 0.5 mcg/kg/hr to minimize agitation in the setting of developmental delay (Tables 1 and 2). Total postoperative opioid administration included two doses of 0.05 mg/kg morphine. Mean pain scores were low. Parents had no concerns regarding pain control.

## 3. Discussion

Adequate pain control for pediatric cardiac surgery is important both *prima facie* because agitation may result in displacement of vital lines, tubes, drains, and wires. Pain control in pediatric cardiac surgery may be particularly challenging as these patients frequently require extended hospital courses, multiple reoperations, and prolonged opioid exposure, resulting in increased medication tolerance.

The ESPB has been explored as an emerging technique in both adult and pediatric cardiac surgery. The vast majority of reports include either single-shot techniques or placement of catheters for continuous postoperative analgesia. Placement of continuous nerve block catheters may be technically challenging, are prone to dislodging, and may present a dilemma in the presence of coagulopathy or anticoagulation needs. An additional possible concern may be pressure injuries caused by catheters during long procedures and especially in the setting of low-perfusion states [8–10]. For these reasons, among others, single-shot techniques are advantageous.

Liposomal bupivacaine is an attractive alternative to plain bupivacaine as it has a longer duration of action due to the extended-release formulation. As a novel drug, the body of evidence regarding liposomal bupivacaine is still under development, with even fewer studies in pediatric populations. Currently, FDA-approved indications for liposomal bupivacaine include interscalene nerve blocks and tissue infiltration, both in adult patients. However, our institution has had success with the use of liposomal bupivacaine for infiltration, plane blocks, and peripheral blocks in pediatric patients. The use of liposomal bupivacaine for ESPB has been reported in adult cardiac surgery and adult breast surgery [11, 12].

In this case series, we report three patients who received single-shot ESPB with liposomal bupivacaine for pediatric cardiac surgery. In all cases, blocks were rapid to perform (approximately 10 min) and technically achievable. No complications of block placement, drug administration, or local anesthetic toxicity were observed. Both patients that underwent sternotomy had good pain control with minimal pain reported after removal of chest tubes on POD2, and neither patient received any opioids during hospitalization after chest tube removal. The patient that underwent thoracotomy incision had minimal pain throughout their entire postoperative period. While a rigorous statistical comparison was not feasible for this case report with historical controls, in a previous study in our institution, Stuth et al. reported a median value of required morphine equivalents for stage 2 palliation of 0.5 mg/kg IV morphine for the first 12 postoperative hours [13]. A more recent study reported the opioid requirements of postoperative pediatric cardiac surgical patients for two consecutive years. The average IV morphine equivalents on POD1 were 2.2/2.17 mg/kg/day (2017/2018), and the average for the first five postoperative days was 0.86/0.62 mg/kg/day (2017/2018) [14].

Although the admixtures and volumes for the patients were highly variable, the patients received similar amounts of local anesthetic by weight, as shown in Table 2. Plain bupivacaine was added to the admixture both to speed block onset of action for incision and to achieve satisfactory spreading in the erector spinae plane.

We chose to report total morphine dose, FLACC pain scores (Face, Leg, Activity, Cry, and Consolability), and RASS scores (Richmond Agitation Sedation Scale). Our findings include moderate opioid use in POD0-1, but negative average RASS scores in those same time periods. While multiple interpretations may be possible, the authors acknowledge that opioids may be used to target multiple goals in the early postop period, especially while many invasive lines, drains, and pacing wires are in place. A negative RASS score correlates to an overall sedated state. After chest tube removal, both sternotomy patients had low pain scores and RASS scores approaching 0, suggestive of awake, comfortable, and a state of calm wakefulness with neither agitation nor sedation.

The authors conclude that, as suggested in previous studies, ESPB may be an attractive option for pain control in pediatric cardiac surgery. No adverse effect was observed, but more extensive studies of the liposomal bupivacaine in the pediatric population would elaborate the safety and pharmacokinetics/pharmacodynamics in these patients.

## Figures and Tables

**Table 1:** Median, 25% and 75%, mean, and standard error (SE) values for the FLACC pain score (Face, Leg, Activity, Cry, and Consolability) and RASS score (Richmond Agitation Sedation Scale). Ranges of FLACC scores observed were 0 to 10 in total. Ranges of RASS scores observed were −5 to +4. The comments column details important clinical events that could contribute to pain scores. Morphine was the sole opioid administered postoperatively in all cases.

	FLACC (Face, Leg, Activity, Cry, and Consolability)				RASS (Richmond Agitation Sedation Scale)				Morphine	Comments
Case 1	Median	25%/75%	Mean	SE	Median	25%/75%	Mean	SE	mg/kg/day	Comments
POD 0	0	0/0	0.61	0.42	−4.5	−5/−4	−3.94	0.37	0.4	Dexmed infusion @ 0.6 mcg/kg/hr
POD 1	0	0/0	0.83	0.34	0	−1/0	−0.21	0.16	0.3	Toradol extubated in the morning
POD 2	0	0/0	0.29	0.02	−1	−1/0	0.29	0.10	0	Chest tubes were removed
POD 3	0	0/0	0.00	0.00	0	0/0	−0.08	0.01	0	—

Case 2	Median	25%/75%	Mean	SE	Median	25%/75%	Mean	SE	Morphine	Comments
POD 0	0	0/3	1.95	0.73	−3	−3/−1	−1.62	0.46	0.46	Dexmed infusion @ 0.6 mcg/kg/hr
POD 1	0	0/0	1.27	0.53	0	0/0	−0.18	0.22	0.28	Toradol, extubated in the morning
POD 2	0	0/0	0.08	0.08	0	0/0	0.00	0	0	Chest tubes were removed
POD 3	0	0/0	0.00	0	0	0/0	0.00	0	0	—

Case 3	Median	25%/75%	Mean	SE	Median	25%/75%	Mean	SE	Morphine	Comments
POD 0	0	0/0	0.09	0.73	0	−3/−1	−1.86	0.23	0.11	Dexmed infusion @ 0.5 mcg/kg/hr. Scheduled tylenol/toradol
POD 1	0	0/0	0.42	0.53	0	0/0	−0.21	0.08	0	Chest tubes removed
POD 2	0	0/0	0.00	0.08	0	0/0	0.00	0	0	—

**Table 2:** A summary of the local anesthetic volume (ml) and dosage (mg/kg) used for each case is presented. The regular bupivacaine referred in the table was added to the liposomal bupivacaine to create the admixture used for the injection.

	Weight (kg)	LB (ml)	LB (mg/kg)	Bupivacaine	Bupivacaine mg/kg
Case 1	7.9	2	3.4 mg/kg	6 ml - 0.25%	1.9 mg/kg
Case 2	27.3	8	3.9 mg/kg	12 ml - 0.375%	1.65 mg/kg
Case 3	19.7	6	4 mg/kg	4 ml - 0.5%	2 mg/kg

## Data Availability

The results of the statistical analysis of the data used to support the findings of this study are included within the article. The raw data including FLACC and RASS scores as well as the analytical methods used may be deidentified for patient name and released upon contacting the corresponding author.

## Abbreviations

ESPB: Erector Spinae Plane Block
AV canal: Atrioventricular canal
POD: Postoperative day
T: Thoracic
PA band: Pulmonary artery band
IV: Intravenous
RV-PA conduit: Right ventricle to pulmonary artery conduit
CHARGE syndrome: Coloboma, Heart defects, Atresia choanae, Growth retardation, Genital abnormalities, and Ear abnormalities syndrome
FLACC pain scale: Face, Leg, Activity, Cry, and Consolability
RASS score: Richmond agitation sedation scale.
